# OTUB1 stabilizes PRRSV matrix protein through a non-canonical deubiquitination mechanism to promote viral replication

**DOI:** 10.1128/jvi.01868-25

**Published:** 2026-03-31

**Authors:** Benjin Liu, Wenqi Liang, Xingyu Li, Shan Jiang, Ziqi Shi, Nian Liu, Rongsheng Zhu, Xuanzhi Dong, Han Zhou, Bin Zhou, Jin Cui

**Affiliations:** 1 College of Veterinary Medicine, Northeast Agricultural University 12430, Harbin, China; University of Kentucky College of Medicine, Lexington, Kentucky, USA

**Keywords:** PRRSV, OTUB1, matrix protein, ubiquitination, deubiquitinase

## Abstract

Porcine reproductive and respiratory syndrome virus (PRRSV) poses a major threat to the swine industry worldwide. The viral matrix (M) protein is essential for virion assembly and infectivity. In this study, we found that the M protein is degraded through the ubiquitin-proteasome system. Immunoprecipitation and mass spectrometry identified the host deubiquitinase OTUB1 as an M-binding partner. Overexpression of OTUB1 increased M protein stability and expression levels, while OTUB1 knockdown promoted M degradation. OTUB1 interacted with the C-terminal ectodomain of the M protein and specifically removed K48-linked ubiquitin chains that target proteins for proteasomal degradation. Mechanistically, a catalytically inactive OTUB1 mutant (C91A) retained the ability to stabilize and deubiquitinate M, while a mutant defective in the non-canonical pathway (D88A) lost this function, indicating that OTUB1 acts primarily by sequestering E2 ubiquitin-conjugating enzymes. Furthermore, we identified UBE2D2 as the specific E2 ubiquitin-conjugating enzyme sequestered by OTUB1 to prevent M protein degradation. Depletion of OTUB1 significantly attenuated PRRSV replication in MARC-145 cells and primary porcine alveolar macrophages. OTUB1 specifically targeted the M protein without stabilizing other PRRSV proteins. Importantly, OTUB1-mediated stabilization was conserved across M proteins from diverse PRRSV strains, including PRRSV-1, PRRSV-2, and emerging NADC30-like and NADC34-like variants. Collectively, our findings revealed a host mechanism exploited by PRRSV to enhance replication and identified OTUB1 as a potential target for antiviral development.

**IMPORTANCE:**

Porcine reproductive and respiratory syndrome (PRRS) poses a substantial threat to global swine production, resulting in major economic losses. The causative agent, PRRS virus (PRRSV), exhibits high genetic variability, which frequently enables it to evade existing vaccine-induced immunity. Our study identifies the host deubiquitinase OTUB1 as a critical factor exploited by PRRSV to stabilize the essential M protein by sequestering the E2 ubiquitin-conjugating enzyme UBE2D2. This mechanism facilitates enhanced viral replication and is conserved across diverse PRRSV strains. Our findings reveal a key host-pathogen interaction and suggest that disrupting OTUB1-mediated stabilization of the M protein may pave the way for novel antiviral strategies against diverse PRRSV variants. This work thus provides a conceptual foundation for future host-directed interventions against this economically significant pathogen.

## INTRODUCTION

Porcine reproductive and respiratory syndrome (PRRS) represents one of the most economically devastating diseases affecting the global swine industry. The disease is characterized by reproductive failure in sows and respiratory distress in pigs of all ages (1). Since its initial emergence in the late 1980s, PRRS has continued to cause substantial economic losses worldwide (2–4). The causative agent, PRRS virus (PRRSV), is an enveloped, positive-sense single-stranded RNA virus with a genome of approximately 15 kb. A hallmark of PRRSV is its remarkable genetic variability, driven by high mutation and recombination rates, which has led to the emergence of diverse strains such as highly pathogenic PRRSV, NADC30-like, and NADC34-like variants, complicating epidemic control (5, 6). The lack of broadly effective vaccines underscores the urgent need for novel intervention strategies targeting key host-virus interactions (7–9).

The PRRSV genome encodes both replicase polyproteins (ORF1a and ORF1b) and multiple structural proteins. The replicase polyproteins are cleaved into non-structural proteins (nsps), several of which have been shown to antagonize host innate immune responses by suppressing interferon production (10–14). Among the structural proteins, the matrix (M) protein plays an essential role in the viral life cycle. It forms a disulfide-linked heterodimer with the major envelope glycoprotein GP5, facilitating viral assembly, entry, and budding (15–18). Co-expression of M and GP5 in vaccine formulations elicits stronger neutralizing antibody responses than either protein alone, highlighting its immunogenic importance (19). Recent studies further indicate that post-translational modifications, such as palmitoylation, are critical for M protein function and viral replication (20). However, the mechanisms regulating M protein stability, particularly through ubiquitination, remain poorly understood.

Ubiquitination is a key post-translational modification that targets lysine residues and participates in diverse cellular processes, such as protein degradation, signal transduction, and immune regulation (21). The ovarian tumor (OTU) protease family consists of cysteine proteases that reverse ubiquitination to regulate cellular signaling pathways (22). OTUB1, a prominent OTU family member, regulates ubiquitination through two distinct mechanisms. The canonical pathway involves direct hydrolysis of ubiquitin chains, with preference for K48-linked and K63-linked polyubiquitin (23). In contrast, the non-canonical mechanism relies on OTUB1 binding to E2 ubiquitin-conjugating enzymes, which blocks ubiquitin transfer (24). OTUB1 also modulates protein ubiquitination through intermediary roles, such as promoting GRAIL deubiquitination via USP8 (25). Previous studies have linked OTUB1 to various physiological processes, including osteoblast differentiation through FGFR2 stabilization (26) and innate immune regulation via deubiquitination of RIG-I and TRAF6 (27, 28). During influenza virus infection, OTUB1 enhances viral replication by stabilizing the NS2 protein (29). Nevertheless, a potential role for OTUB1 during PRRSV infection has not been explored.

This study investigated the regulatory effects of OTUB1 on M protein stability and ubiquitination, and the resulting impact on PRRSV replication. Results demonstrated that M protein is degraded through the ubiquitin-proteasome system (UPS), and that OTUB1 counteracts this process by sequestering the E2 ubiquitin-conjugating enzyme UBE2D2 through a non-canonical deubiquitination mechanism. OTUB1 depletion significantly reduced PRRSV replication, and this activity displayed specificity toward the M protein among other PRRSV-encoded proteins. Furthermore, the stabilizing function of OTUB1 was conserved across M proteins from diverse PRRSV strains. These findings reveal a novel mechanism where PRRSV exploits host deubiquitinase activity to facilitate viral replication, identifying OTUB1 as a potential target for antiviral strategies.

## RESULTS

### Degradation of M protein through UPS

**Fig 1:**
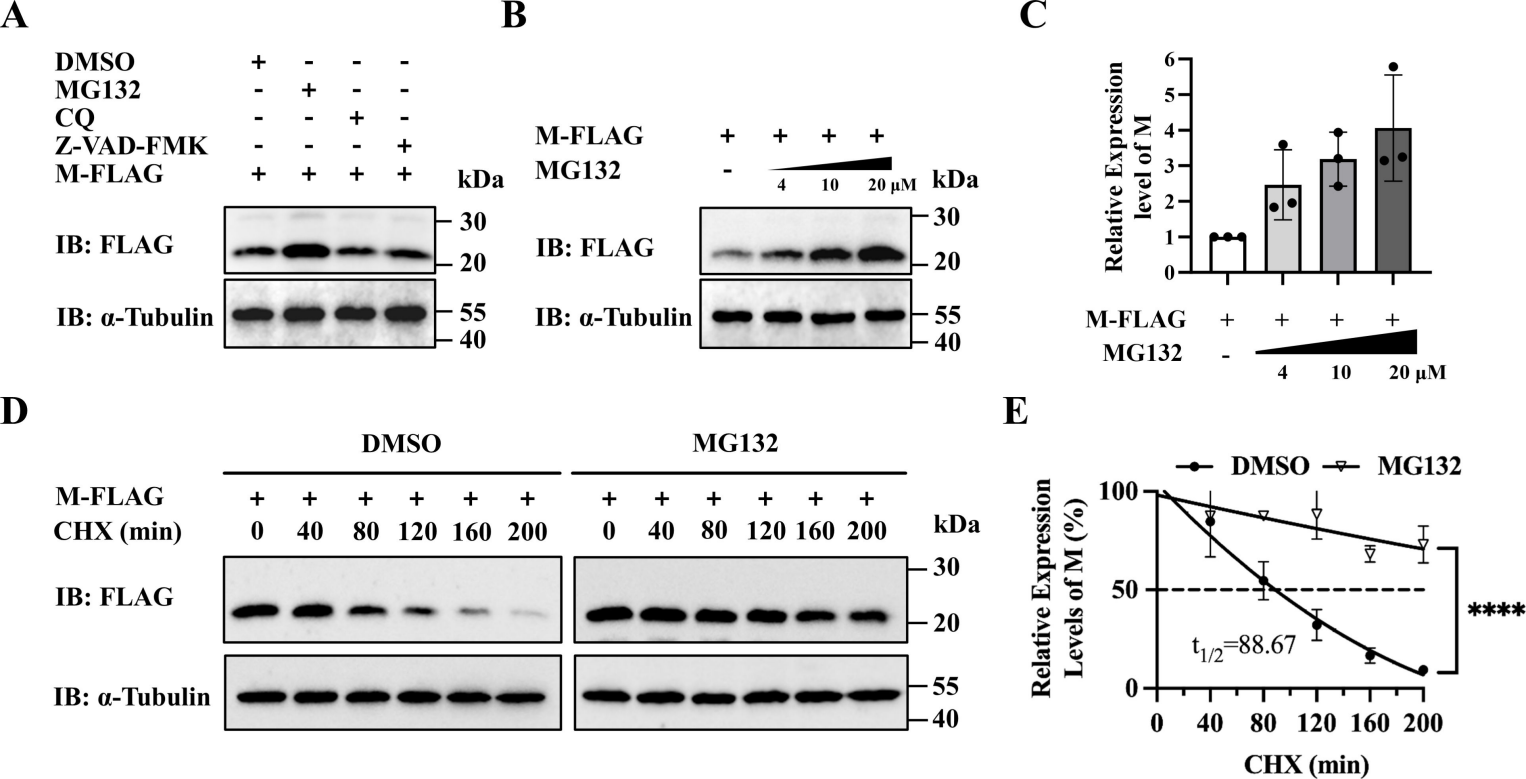
Degradation of the M protein via the UPS. (**A**) Effect of protein degradation inhibitors on M protein stability. HEK-293T cells expressing pFLAG-M were treated with DMSO, MG132 (10 μM), chloroquine (CQ, 50 μM), or Z-VAD-FMK (50 μM) for 10 h. M protein levels were analyzed by Western blot. (**B and C**) Dose-dependent accumulation of the M protein. HEK-293T cells expressing pFLAG-M were treated with increasing concentrations of MG132 (0, 4, 10, and 20 μM) for 10 h. Relative protein abundance was quantified. (**D and E**) CHX chase assay. HEK-293T cells transfected with pFLAG-M were treated with CHX (100 μg/mL) in the presence of DMSO or MG132. M protein levels were monitored at the indicated time points, and the half-life was plotted.

Previous studies have established that ubiquitination regulates multiple PRRSV proteins, including nsp1β, nsp3, nsp4, nsp5, nsp12, GP3, GP4, and GP5 (30–36). The viral non-structural protein nsp2TF has been shown to reduce K48-linked ubiquitination of the M and GP5 proteins through deubiquitination (36). To further examine whether the M protein is degraded specifically via UPS, HEK-293T cells were transfected with pFLAG-M and treated with DMSO, the proteasome inhibitor MG132, autophagy-lysosome inhibitor CQ, and apoptosis inhibitor Z-VAD-FMK. MG132 treatment significantly increased M protein abundance in a dose-dependent manner, whereas no notable changes were observed in the groups treated with autophagy or apoptosis inhibitors (Fig. 1A through C). To further assess degradation kinetics, protein half-life was determined using cycloheximide (CHX) chase assays. In the DMSO-treated control group, the M protein was rapidly degraded, exhibiting a short half-life. In contrast, MG132 treatment markedly enhanced protein stability and prolonged its half-life (Fig. 1D and E). These results confirm that the M protein is targeted for degradation through UPS. To explore host factors involved in this regulation, immunoprecipitation followed by liquid chromatography-mass spectrometry was performed (Fig. S1A). While pathway analysis broadly indicated the involvement of protein degradation processes (Fig. S1B), the deubiquitinase OTUB1 was identified as a putative-binding partner, with its presence in the M protein immunoprecipitates confirmed by unique peptide spectral analysis (Fig. S1C).

### OTUB1 enhances the half-life and expression level of M protein

**Fig 2:**
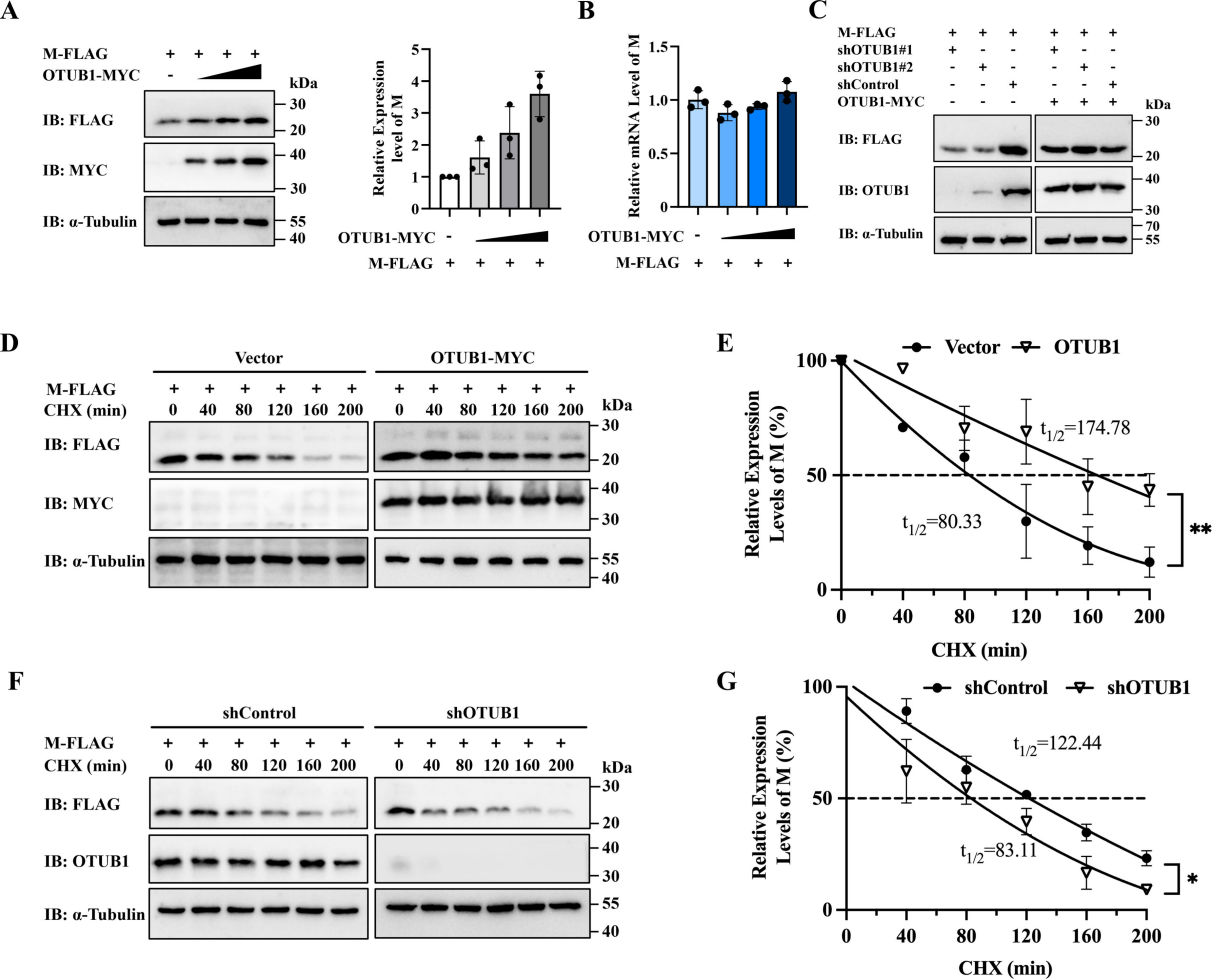
OTUB1 enhances M protein abundance by extending its half-life. (**A**) Western blot analysis of M protein levels in HEK-293T cells co-transfected with pFLAG-M and increasing amounts of pMYC-OTUB1. (**B**) RT-qPCR quantification of M protein mRNA levels in cells. (**C**) Rescue of M protein expression. Stable OTUB1-knockdown (shOTUB1) or control (shControl) HEK-293T cells were co-transfected with pFLAG-M and either pMYC-OTUB1 or empty vector (EV). (**D and E**) M protein half-life analysis upon OTUB1 overexpression. HEK-293T cells co-expressing pFLAG-M and pMYC-OTUB1 (or EV) were treated with CHX and analyzed at the indicated time points. (**F and G**) M protein half-life analysis in OTUB1-knockdown cells. Stable shOTUB1 or shControl HEK-293T cells were transfected with pFLAG-M and subjected to CHX chase assays.

To validate the regulatory role of OTUB1, HEK-293T cells were co-transfected with pFLAG-M and increasing amounts of pMYC-OTUB1. Western blot analysis revealed that M protein abundance increased in a dose-dependent manner, correlating with elevated OTUB1 expression levels (Fig. 2A). To determine whether this accumulation resulted from transcriptional upregulation, M protein mRNA levels were quantified by RT-qPCR. No significant change was observed, indicating that OTUB1 regulates the M protein at the post-translational level (Fig. 2B). To corroborate this regulatory relationship, stable OTUB1-knockdown HEK-293T cell lines were established. Upon transfection with pFLAG-M, a marked reduction in M protein expression was observed in OTUB1-depleted cells. Crucially, this reduction was rescued by OTUB1 complementation, confirming that OTUB1 positively regulates M protein expression (Fig. 2C). To further assess protein stability, CHX chase assays were performed. Results demonstrated that OTUB1 overexpression significantly prolonged the half-life of the M protein, whereas OTUB1 knockdown accelerated its degradation (Fig. 2D through G). Collectively, these data demonstrate that OTUB1 promotes the intracellular accumulation of the M protein by enhancing its stability.

### Interaction and co-localization between OTUB1 and the M protein

**Fig 3:**
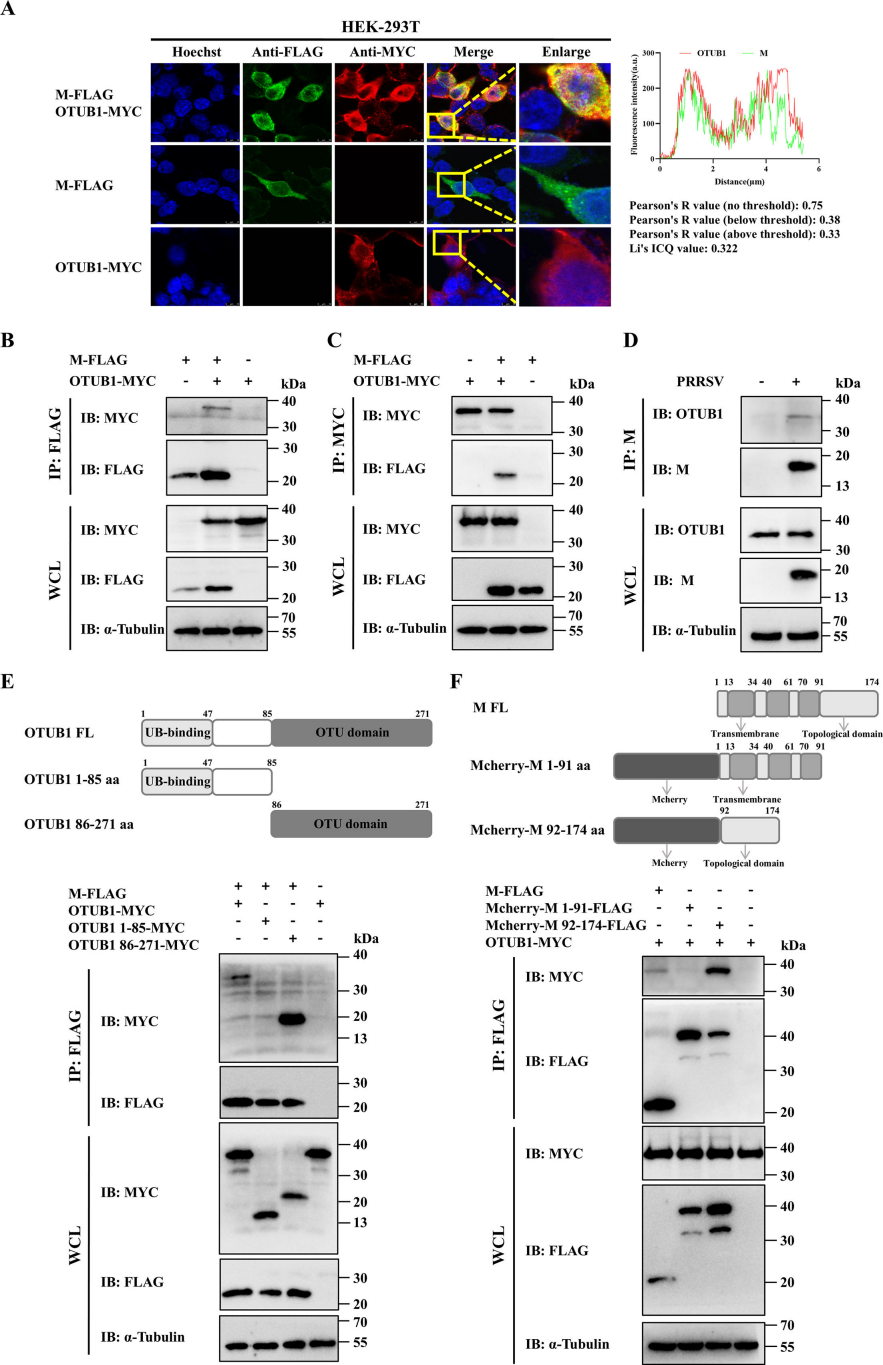
Interaction and co-localization between OTUB1 and the M protein. (**A**) Confocal microscopy showing the co-localization of FLAG-M (green) and MYC-OTUB1 (red) in HEK-293T cells. Nuclei were stained with Hoechst 33342 (blue). (**B and C**) Co-IP analysis of the interaction between OTUB1 and the M protein in HEK-293T cells co-transfected with pFLAG-M and pMYC-OTUB1. (**D**) Endogenous Co-IP assay in PRRSV-infected cells. MARC-145 cells were infected with PRRSV L251 (multiplicity of infection [MOI] = 0.5), and lysates were immunoprecipitated with anti-M antibody. (**E**) Mapping of the OTUB1 domain required for M binding. HEK-293T cells were co-transfected with pFLAG-M and full-length or truncated MYC-OTUB1 (1–85 or 86–271 aa). (**F**) Mapping of the M protein domain required for OTUB1 binding. Cells were co-transfected with pMYC-OTUB1 and full-length or truncated FLAG-M (1–91 or 92–174 aa).

To determine whether OTUB1 associates with the M protein, co-localization and interaction analyses were performed. Confocal fluorescence microscopy of HEK-293T cells co-expressing FLAG-tagged M and MYC-tagged OTUB1 revealed clear co-localization of the two proteins (Fig. 3A). Protein interaction was further investigated through co-immunoprecipitation (Co-IP) assay. Cell lysates from HEK-293T cells co-expressing pFLAG-M and pMYC-OTUB1 were immunoprecipitated with either FLAG or MYC antibodies. Reciprocal pulldowns consistently demonstrated a specific association between OTUB1 and the M protein (Fig. 3B and C). Crucially, to validate this interaction during viral infection conditions, endogenous Co-IP assays were performed in PRRSV-infected MARC-145 cells. The results confirmed that the viral M protein interacts with endogenous OTUB1 during actual infection (Fig. 3D). Subsequently, the molecular domains mediating this interaction were mapped using truncation mutants. Co-IP experiments identified the C-terminal OTU domain of OTUB1 (amino acids 86–271) as the region responsible for binding to the M protein (Fig. 3E). Complementary mapping of the M protein revealed that its C-terminal ectodomain is necessary and sufficient for the interaction, whereas the N-terminal transmembrane region was dispensable (Fig. 3F).

### OTUB1 stabilizes the M protein by removing K48-linked ubiquitin chains

**Fig 4:**
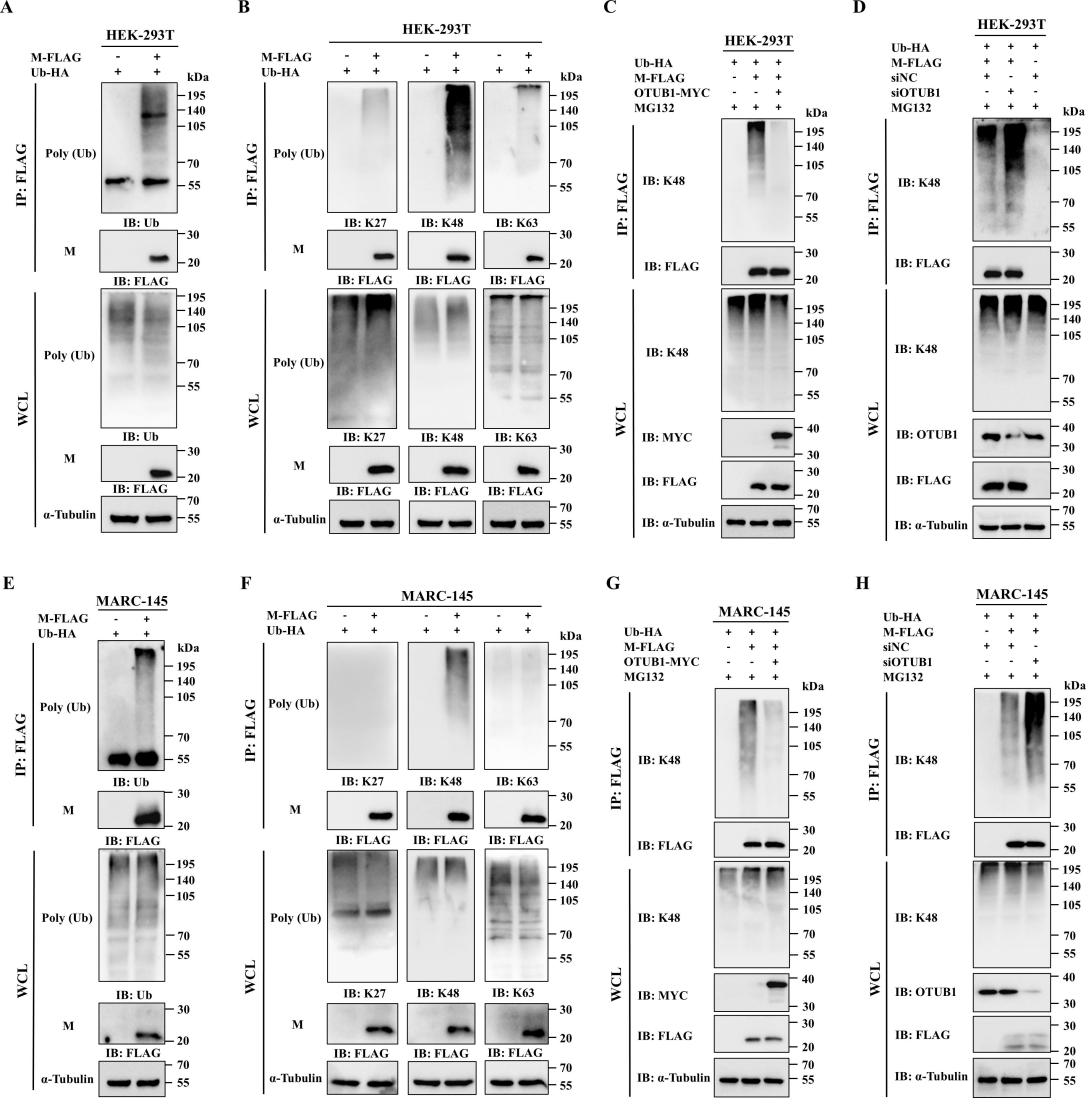
OTUB1 specifically removes K48-linked ubiquitin chains from the M protein. (**A**) Ubiquitination assay of the M protein. HEK-293T cells co-transfected with pFLAG-M and pHA-Ub were subjected to IP with anti-FLAG antibody, followed by Western blot with a pan-ubiquitin antibody. (**B**) Determination of ubiquitin linkage types. The immunoprecipitates were analyzed using linkage-specific antibodies against K27-linked, K48-linked, or K63-linked ubiquitin chains. (**C and D**) Effect of OTUB1 overexpression (**C**) and knockdown (**D**) on K48-linked ubiquitination. HEK-293T cells were transfected as indicated, and the levels of K48-linked ubiquitination on the M protein were detected using a K48 linkage-specific antibody. (**E–H**) Validation in MARC-145 cells. Experiments corresponding to panels A to D were repeated in MARC-145 cells. M protein ubiquitination (**E**) and linkage specificity (**F**) were verified using the respective antibodies. The regulatory effects of OTUB1 overexpression (**G**) and knockdown (**H**) on K48-linked ubiquitination were assessed using a K48 linkage-specific antibody. In panels C, D, G, and H, cells were treated with MG132 (10 μM) before harvesting.

To elucidate the ubiquitination profile of the M protein, HEK-293T cells co-transfected with pFLAG-M and pHA-Ub were treated with MG132 to enrich ubiquitinated proteins. Subsequent immunoprecipitation with anti-FLAG antibody followed by immunoblotting with a pan-ubiquitin antibody confirmed robust ubiquitination of the M protein (Fig. 4A). Probing these immunoprecipitates with linkage-specific antibodies revealed that M protein underwent robust K48-linked polyubiquitination, whereas the signals for K27-linked and K63-linked chains were negligible. This identifies K48-linked chains as the primary ubiquitin signal conjugated to the M protein (Fig. 4B). Assessing the regulatory impact of OTUB1 on this linkage specificity revealed that overexpression significantly attenuated K48-linked ubiquitination (Fig. 4C), whereas knockdown markedly enhanced the phenotype (Fig. 4D). To substantiate these observations in PRRSV-susceptible cells, identical transfection assays were performed in MARC-145 cells (Fig. 4E through H). Consistently, the results obtained in MARC-145 cells corroborated the HEK-293T findings, confirming that the M protein is primarily targeted by K48-linked ubiquitination and that OTUB1 effectively antagonizes K48-mediated degradation.

### OTUB1 stabilizes the M protein by sequestering UBE2D2

**Fig 5:**
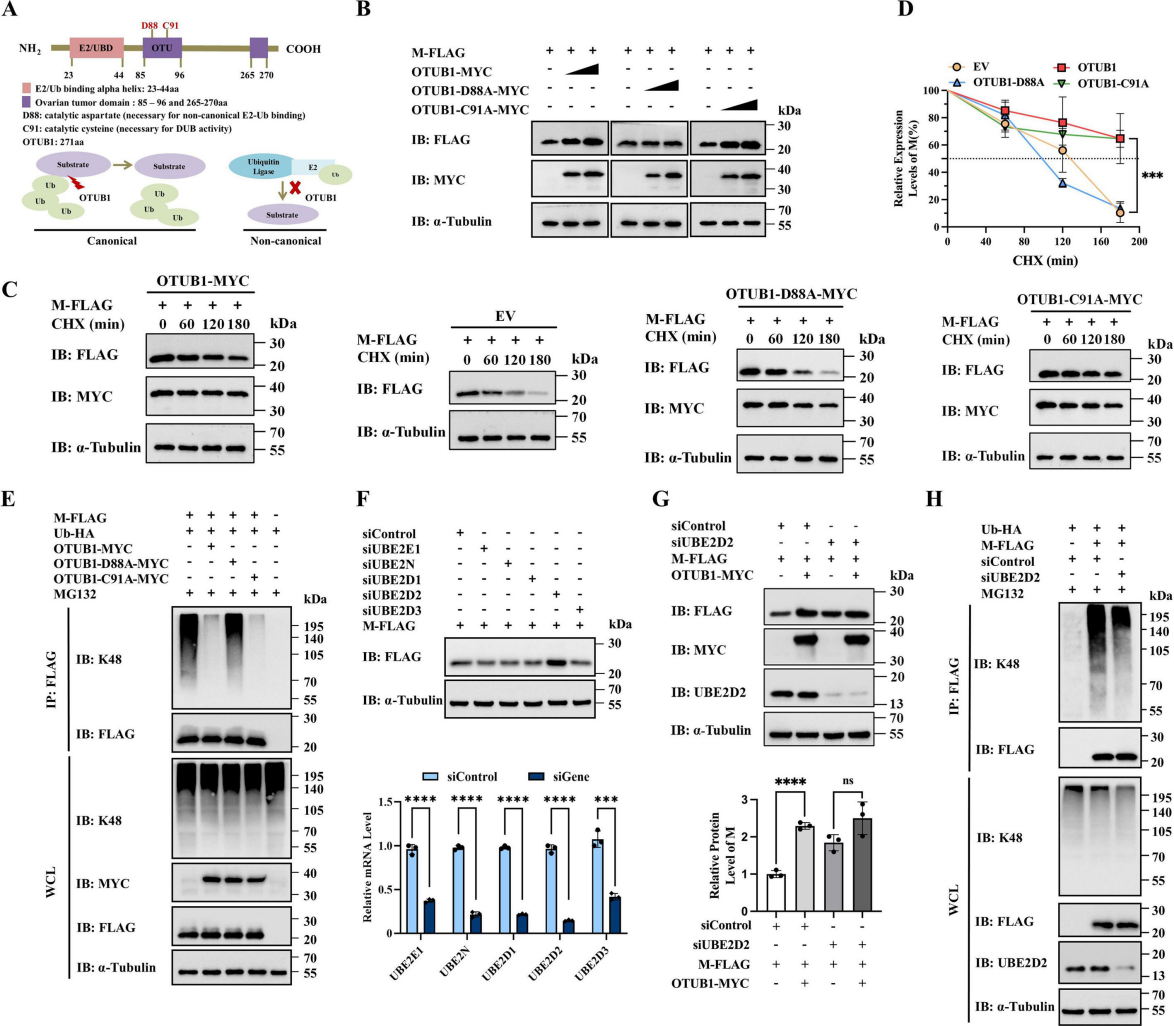
OTUB1 stabilizes the M protein by sequestering UBE2D2. (**A**) Schematic representation of OTUB1 functional domains and the mutants used (C91A: catalytically inactive; D88A: E2-binding deficient). (**B**) Western blot showing M protein levels in HEK-293T cells co-transfected with WT, C91A, or D88A OTUB1. (**C and D**) CHX chase assays comparing the M protein half-life in the presence of WT or mutant OTUB1. (**E**) Effect of WT and mutant OTUB1 on K48-linked ubiquitination of the M protein. (**F**) Screening of E2 enzymes. HEK-293T cells were transfected with siRNAs targeting multiple E2 enzymes or a control siRNA, and M protein accumulation was analyzed. (**G**) Dependency on UBE2D2. HEK-293T cells were co-transfected with OTUB1 and M protein in the presence of siUBE2D2 or siControl. The knockdown of UBE2D2 was verified by Western blot. The bar graph shows the quantification of M protein band intensities relative to α-Tubulin from three independent experiments. (**H**) Effect of UBE2D2 knockdown on K48-linked ubiquitination of the M protein. UBE2D2 protein levels in WCL were also detected.

OTUB1 regulates substrate ubiquitination through two well-characterized mechanisms. The canonical pathway involves direct catalytic hydrolysis of ubiquitin chains, relying on a conserved catalytic triad (C91, H265, and D267), where mutations such as C91A abolish enzymatic activity (37–39). In contrast, the non-canonical mechanism functions by sequestering E2 ubiquitin-conjugating enzymes through the N-terminal domain, a process strictly dependent on residue D88 (Fig. 5A) (40, 41).

To distinguish which mechanism governs M protein stabilization, we compared the effects of wild-type (WT) OTUB1, the catalytically inactive mutant (C91A), and the non-canonical binding-deficient mutant (D88A) in HEK-293T cells. Western blot analysis revealed that both WT OTUB1 and the C91A mutant significantly increased M protein levels. In contrast, the D88A mutant failed to stabilize the M protein (Fig. 5B). Consistently, CHX chase assays confirmed that WT and C91A OTUB1 extended the half-life of the M protein, whereas the D88A mutant exhibited no such effect (Fig. 5C and D). Furthermore, analysis of K48-linked ubiquitination demonstrated that WT and C91A OTUB1 effectively suppressed ubiquitination, while the D88A mutant failed to reduce M protein ubiquitination (Fig. 5E). Collectively, these data indicate that OTUB1 prevents M protein degradation primarily through the non-canonical inhibition of E2 ubiquitin-conjugating enzymes, rather than through direct catalytic deubiquitination.

To identify the specific E2 enzyme mediating M protein regulation, we conducted a targeted siRNA-based screen against a panel of known OTUB1-interacting E2s (40, 42). Among the candidates tested, silencing of UBE2D2 resulted in a significant accumulation of the transiently expressed M protein (Fig. 5F). To validate the functional dependency between OTUB1 and UBE2D2, we assessed the stabilizing effect of OTUB1 under UBE2D2-depleted conditions. The knockdown efficiency of UBE2D2 was successfully confirmed at the protein level by Western blot analysis. As shown in Fig. 5G, the enhancement of M protein abundance by OTUB1 was markedly reduced when UBE2D2 was knocked down. Quantification of M protein band intensities from three independent experiments confirmed this significant reduction, indicating that OTUB1 relies on UBE2D2 to exert its protective function. Furthermore, we examined the impact of UBE2D2 depletion on the ubiquitination status of the M protein. Co-immunoprecipitation assays demonstrated that silencing UBE2D2 substantially attenuated the K48-linked ubiquitination of the M protein (Fig. 5H), confirming UBE2D2 as the E2 ligase responsible for K48-linked polyubiquitination. Collectively, these data provide robust evidence that OTUB1 stabilizes the M protein primarily by sequestering UBE2D2, consequently blocking the formation of K48-linked ubiquitin chains.

### OTUB1 knockdown impairs PRRSV replication

**Fig 6:**
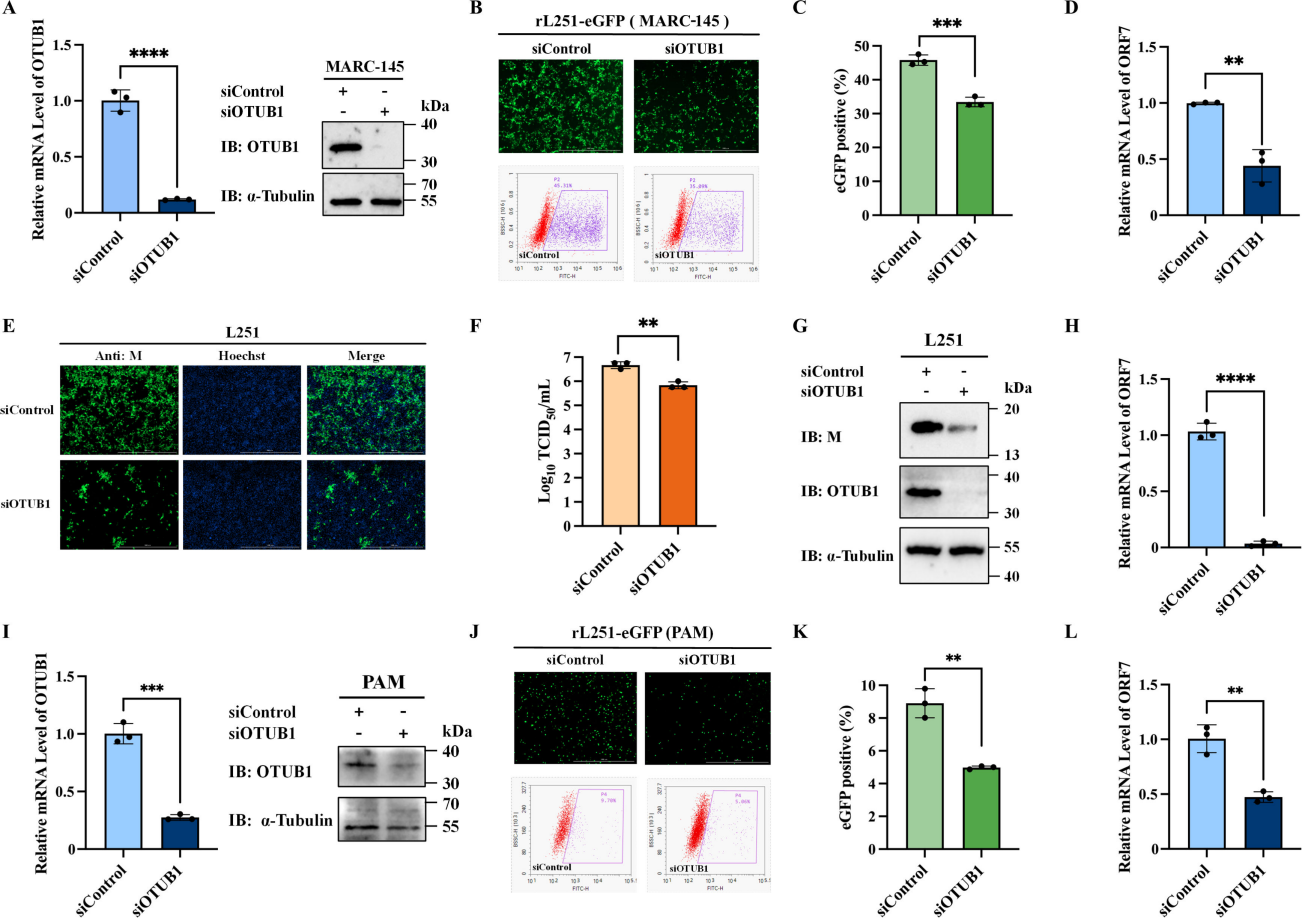
OTUB1 knockdown impairs PRRSV replication in permissive cells. (**A**) Verification of OTUB1 knockdown efficiency in MARC-145 cells by RT-qPCR and Western blot. (**B and C**) Effect of OTUB1 knockdown on rL251-eGFP replication. MARC-145 cells transfected with siOTUB1 were infected with rL251-eGFP (MOI = 0.5). Viral replication was assessed by fluorescence microscopy (**B**) and flow cytometry (**C**) at 36 hpi. (**D**) RT-qPCR quantification of viral mRNA levels from cells in panel B. (**E–H**) Effect of OTUB1 knockdown on WT PRRSV replication. MARC-145 cells transfected with siOTUB1 were infected with L251 (MOI = 0.5). Viral replication was assessed at 36 hpi by IFA (**E**), TCID50 (**F**), Western blot (**G**), and RT-qPCR (**H**). (**I**) Verification of OTUB1 knockdown in primary PAMs. (**J–L**) Effect of OTUB1 knockdown on PRRSV replication in PAMs. Cells were infected with rL251-eGFP (MOI = 0.5) and analyzed by fluorescence microscopy (**J**), flow cytometry (**K**), and RT-qPCR (**L**).

To investigate the functional significance of OTUB1 in PRRSV replication, OTUB1-specific siRNAs were transfected into MARC-145 cells. Efficient silencing of OTUB1 was confirmed by RT-qPCR and Western blot analysis at 48 h post-transfection (Fig. 6A). Subsequently, cells were infected with the reporter virus rL251-eGFP at an MOI of 0.5. Fluorescence microscopy at 36 h post-infection visualized substantially reduced fluorescence intensity in OTUB1-depleted cells. Flow cytometric analysis confirmed a significant decrease in the proportion of eGFP-positive cells compared to control-transfected cells (Fig. 6B and C). Consistently, RT-qPCR analysis indicated a significant decrease in viral mRNA level following OTUB1 knockdown (Fig. 6D). Parallel results were obtained with the wild-type PRRSV strain L251 (MOI = 0.5). Immunofluorescence staining using an anti-M antibody showed diminished signal intensity in OTUB1-knockdown cells (Fig. 6E). Viral titers in culture supernatants were significantly reduced compared to control conditions (Fig. 6F). Both viral mRNA and protein levels were substantially decreased upon OTUB1 depletion (Fig. 6G and H). In addition, primary porcine alveolar macrophages (PAMs) were isolated and subjected to OTUB1 knockdown. Effective reduction of OTUB1 expression was verified at 24 h post-transfection (Fig. 6I). Following infection with rL251-eGFP (MOI = 0.5), OTUB1-silenced PAMs exhibited weakened fluorescence and a significantly lower percentage of eGFP-positive cells compared to controls (Fig. 6J and K). RT-qPCR analysis further confirmed a significant reduction in viral mRNA levels (Fig. 6L). Collectively, these data demonstrated that OTUB1 depletion effectively restricts PRRSV replication.

To assess whether elevated OTUB1 levels promote viral growth, a stable OTUB1-overexpressing MARC-145 cell line was generated (Fig. S2A and B). However, infection assays with the reporter virus (MOI = 0.5) indicated that OTUB1 overexpression did not significantly enhance viral replication (Fig. S2C). Furthermore, RT-qPCR and virus titration analysis of WT L251 replication in MARC-145-OTUB1 cells showed no significant promotion of viral proliferation (Fig. S2D and E). Finally, to examine OTUB1 expression dynamics during viral infection, MARC-145 cells infected with PRRSV (MOI = 0.5) were analyzed by qPCR and Western blot at 12, 24, and 36 hpi. The results indicated that OTUB1 levels remained unchanged at both mRNA and protein levels throughout the course of infection (Fig. S2F and G).

### OTUB1 specifically regulates the PRRSV M protein

**Fig 7:**
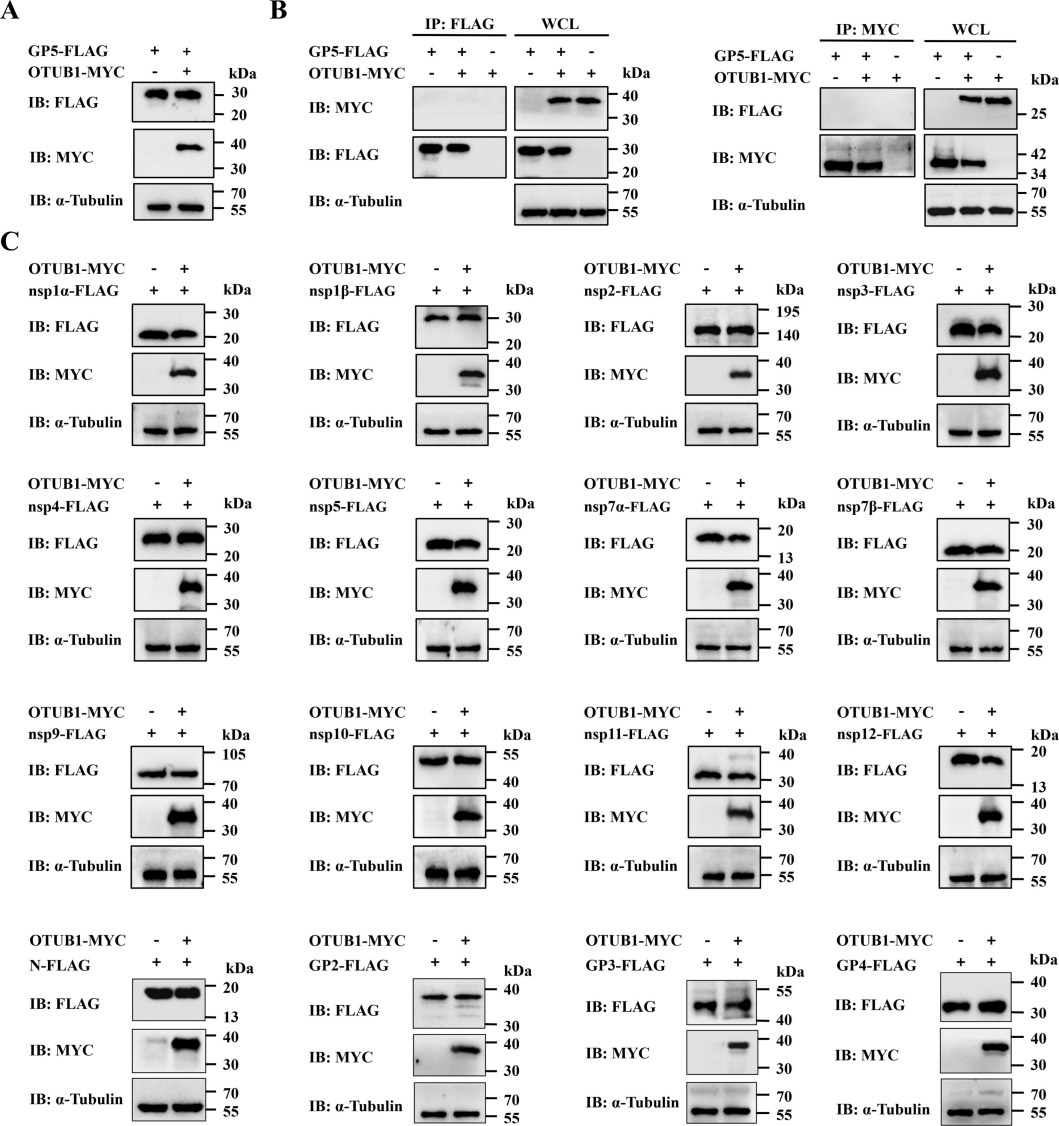
Specificity of OTUB1 toward the M protein. (**A**) Western blot analysis of GP5 protein levels in HEK-293T cells co-transfected with pFLAG-GP5 and pMYC-OTUB1. (**B**) Co-IP analysis showing the lack of interaction between OTUB1 and GP5. (**C**) Specificity screening. HEK-293T cells were co-transfected with pMYC-OTUB1 and plasmids encoding various PRRSV structural proteins (N, GP2, GP3, and GP4) or 12 non-structural proteins.

To assess the substrate specificity of OTUB1 beyond the M protein, initial investigation focused on the envelope glycoprotein GP5. GP5 not only forms a disulfide-bonded heterodimer with the M protein but is also known to be degraded through UPS (15, 36). However, in co-transfection experiments using HEK-293T cells, OTUB1 overexpression did not enhance GP5 protein levels (Fig. 7A). Consistently, co-immunoprecipitation assay detected no interaction between OTUB1 and GP5 (Fig. 7B). This finding distinguishes OTUB1 from the viral nsp2TF protein, which exhibits deubiquitinating activity toward both GP5 and M proteins (36), indicating that GP5 is not recognized as a substrate by OTUB1. To further rule out potential effects on other viral components, the regulation of additional PRRSV proteins was comprehensively evaluated. HEK-293T cells were co-transfected with pMYC-OTUB1 and plasmids encoding 12 distinct non-structural proteins as well as other structural proteins, including the nucleocapsid (N) protein and glycoproteins GP2, GP3, and GP4. Western blot analysis confirmed that none of these viral proteins showed enhanced stability following OTUB1 overexpression (Fig. 7C). Collectively, these findings established the high specificity of OTUB1 toward the M protein, with no detectable regulatory effect on other PRRSV-encoded proteins.

### OTUB1 stabilizes M proteins across diverse PRRSV subtypes

**Fig 8:**
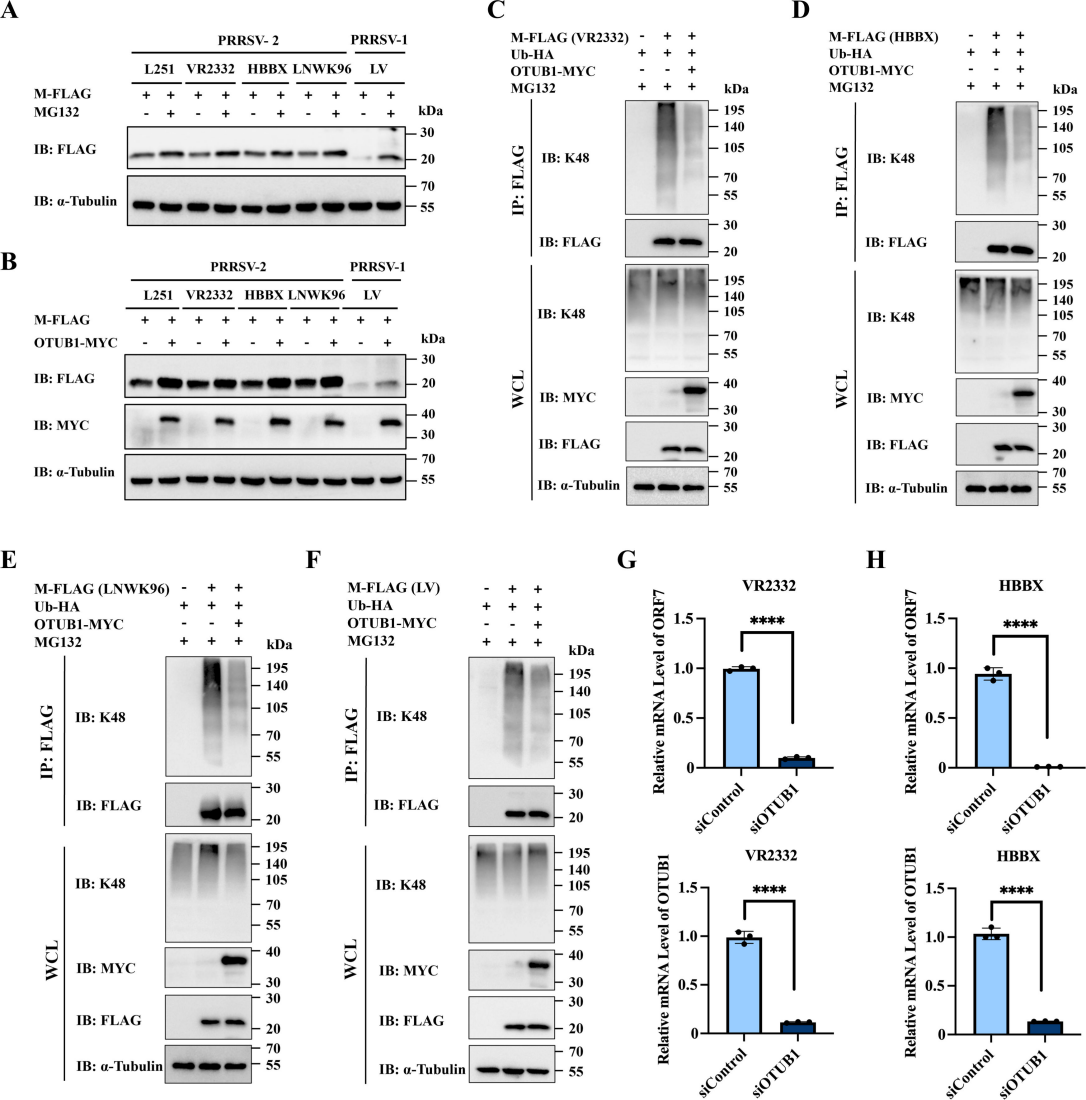
OTUB1-mediated stabilization of the M protein is conserved across diverse PRRSV genotypes. (**A**) Effect of MG132 on the stability of M proteins from PRRSV-1 (LV), PRRSV-2 (VR2332), NADC30-like (HBBX), and NADC34-like (LNWK96) strains. (**B**) Effect of OTUB1 overexpression on the abundance of M proteins from the indicated PRRSV strains. (**C–F**) Effect of OTUB1 on K48-linked ubiquitination of M proteins from VR2332 (**C**), HBBX (**D**), LNWK96 (**E**), and LV (**F**). (**G and H**) Effect of OTUB1 knockdown on the replication of VR2332 (**G**) and HBBX (**H**) strains in MARC-145 cells. Viral mRNA levels were quantified by RT-qPCR. In all ubiquitination assays, cells were treated with MG132 before harvesting.

To evaluate whether OTUB1 exhibits broad-spectrum regulatory effects across diverse PRRSV lineages, a comparative analysis of M proteins from representative viral subtypes was performed. Eukaryotic expression vectors were constructed to encode M proteins derived from PRRSV-1 strain LV, PRRSV-2 classical strain VR2332, as well as the prevalent NADC30-like (HBBX) and NADC34-like (LNWK96) strains. Upon transfection into HEK-293T cells, MG132 treatment significantly enhanced the expression of each M protein variant, confirming UPS-mediated degradation is a conserved feature of the M protein across PRRSV genotypes (Fig. 8A). Furthermore, co-transfection assays demonstrated that OTUB1 overexpression significantly increased the abundance of all M proteins (Fig. 8B). Ubiquitination assays further demonstrated that OTUB1 reduced K48-linked ubiquitination of all tested M protein (Fig. 8C through F). Finally, to validate these findings in the context of infection, we silenced OTUB1 in MARC-145 cells and assessed the replication of VR2332 and HBBX strains. qPCR analysis indicated that OTUB1 knockdown significantly reduced the viral mRNA levels for both strains (Fig. 8G and H). Collectively, these findings establish that OTUB1-mediated stabilization of the M protein is a conserved mechanism maintained throughout PRRSV genetic diversity.

## DISCUSSION

PRRSV remains a severe threat to global swine production, with effective control hampered by high viral genetic variability and limited efficacy of existing vaccines (5). The viral M protein forms a disulfide-linked heterodimer with envelope glycoprotein GP5 and is essential for virion assembly (18). High conservation across PRRSV strains makes this protein a promising intervention target (43, 44). Previous work identified viral non-structural protein nsp2TF as a factor that deubiquitinates both M and GP5 to regulate their stability (36), but the role of host-derived DUBs in modulating PRRSV protein stability, especially M protein stability, has not been addressed. This study confirms host deubiquitinase OTUB1 as a key cellular factor exploited by PRRSV to specifically stabilize M protein and uncovers a novel non-canonical molecular mechanism supporting efficient viral replication.

Initial experiments verified PRRSV M protein degradation via the UPS, a core pathway for targeted protein turnover. Immunoprecipitation coupled with liquid chromatography-mass spectrometry identified OTUB1, a ubiquitination-related host protein, among M protein-interacting factors. Subsequent functional validation showed that OTUB1 enhances M protein stability and steady-state expression in a dose-dependent manner, with no detectable changes in M protein mRNA levels. This indicates that OTUB1 regulates M protein abundance at the post-translational level. Further mapping revealed that OTUB1 binds the C-terminal ectodomain of M protein via its own C-terminal OTU domain, highlighting the structural specificity of this host-virus interaction.

A key mechanistic insight emerges from understanding how OTUB1 mediates M protein stabilization. Our data confirm that OTUB1 specifically removes K48-linked ubiquitin chains from the M protein. Crucially, this activity functions independently of the canonical catalytic activity of OTUB1. Mutagenesis studies identified that the catalytically inactive OTUB1 C91A mutant retained full capacity to stabilize M protein and reduce K48-linked ubiquitination, while the E2-binding-deficient OTUB1 D88A mutant lost this function entirely. This distinguishes OTUB1 action from canonical deubiquitination, which involves transient DUB substrate interactions. OTUB1 relies on stable complex formation with M protein to sequester E2 enzymes and block ubiquitin transfer. Subsequently, we identified UBE2D2 as the specific E2 ubiquitin-conjugating enzyme responsible for M protein ubiquitination.

To validate the biological significance of this interaction, we assessed PRRSV replication in both OTUB1-depleted and OTUB1-overexpressing cells. OTUB1 knockdown significantly impaired viral replication in both MARC-145 cells and PAMs, confirming OTUB1 as an essential host dependency factor. Interestingly, OTUB1 overexpression did not further enhance viral replication in permissive cells. This suggests that endogenous OTUB1 levels are likely sufficient to maintain M protein stability, rendering additional overexpression redundant for viral replication. Additionally, the amino acid sequence of OTUB1 is 100% identical among humans, monkeys, and pigs. This high degree of evolutionary conservation is consistent with the broad efficacy of OTUB1 observed in our diverse cell models.

OTUB1 additionally exhibits remarkable substrate specificity among PRRSV-encoded proteins. Unlike nsp2TF, which deubiquitinates both M and GP5, OTUB1 specifically stabilizes the M protein without detectable effects on other structural proteins (GP2, GP3, GP4, GP5, and N) or any of the 12 tested PRRSV non-structural proteins. Such selectivity likely arises from the structural constraints of the OTUB1 non-canonical mechanism that require direct physical interaction with substrates. This specificity may enable PRRSV to precisely regulate the stability of key viral proteins without disrupting the functions of other viral factors.

Critically, OTUB1-mediated M protein stabilization is conserved across diverse PRRSV genotypes, including PRRSV-1, PRRSV-2, and emerging NADC30-like and NADC34-like variants. This conservation identifies a vulnerable point in the PRRSV replication cycle that is not subject to the high genetic variability of viral proteins themselves, supporting the potential for the development of antiviral strategies against diverse PRRSV genotypes targeting the interaction between OTUB1, M protein, and UBE2D2.

In summary, this study reveals that PRRSV hijacks the non-canonical E2-binding function of host deubiquitinase OTUB1 to stabilize the essential M protein by sequestering UBE2D2. This specific interaction exhibits remarkable substrate selectivity and conservation across diverse PRRSV genotypes. These findings establish the OTUB1-M crosstalk as a virus-dependent vulnerability, offering a promising target for developing conserved therapeutic strategies against this economically consequential pathogen.

## MATERIALS AND METHODS

### Cells and viruses

HEK-293T and MARC-145 cells were obtained from the American Type Culture Collection (ATCC, Manassas, VA, USA) and cultured in Dulbecco’s modified Eagle’s medium (DMEM; Gibco, USA) supplemented with 10% fetal bovine serum (FBS; Gibco, USA) and 1% penicillin-streptomycin (Gibco, USA) at 37°C with 5% CO_2_. Primary PAMs were cultured in Roswell Park Memorial Institute 1640 medium (RPMI-1640; Gibco, USA) containing 10% FBS and 2% penicillin-streptomycin. PRRSV strains L251 (GenBank accession no. PV631263) and rL251-eGFP (a recombinant strain expressing enhanced green fluorescent protein) (45), HBBX (GenBank accession no. PP946131.1), and VR2332 (GenBank accession no. EF536003.1) were preserved in our laboratory. Viral propagation was performed in MARC-145 cells using DMEM supplemented with 2% FBS. Viral titers were determined by the Reed-Muench method.

### Antibodies

The following antibodies were used in this study. Rabbit polyclonal antibodies against OTUB1 (for WB, 1:1,000; for IFA, 1:50; catalog no. 10573-1-AP), MYC (for WB, 1:8,000; for IFA, 1:400; catalog no. 16286-1-AP), rabbit monoclonal antibodies against Flag (for WB, 1:20,000; catalog no. 80801-2-RR), mouse monoclonal antibody against Flag (for IFA, 1:800; catalog no. 66008-4-Ig), α-Tubulin (for WB, 1:20,000; catalog no. 66031-1-Ig), horseradish peroxidase (HRP)-conjugated goat anti-rabbit IgG secondary antibody (for WB, 1:8,000; catalog no. RGAR001), HRP-conjugated goat anti-mouse IgG secondary antibody (for WB, 1:8,000; catalog no. RGAM001), Multi-rAb CoraLite Plus 594 recombinant goat anti-rabbit IgG secondary antibody (for IFA, 1:1,000; catalog no. RGAR004), Multi-rAb CoraLite Plus 488 recombinant goat anti-rabbit IgG secondary antibody (for IFA, 1:1,000; catalog no. RGAR002), and Multi-rAb CoraLite Plus 488 recombinant goat anti-mouse IgG secondary antibody (for IFA, 1:1,000; catalog no. RGAM002) were purchased from Proteintech Group Inc. (Wuhan, China). Additionally, the mouse monoclonal anti-Flag antibody specifically used for immunoprecipitation (IP, 1:100; catalog no. AB0008) was obtained from Abways Technology (Shanghai, China). K48 linkage-specific polyubiquitin (D9D5) rabbit monoclonal antibody (for WB, 1:1,000; catalog no. 8081T), K63 linkage-specific polyubiquitin (D7A11) rabbit monoclonal antibody (for WB, 1:1,000; catalog no. 5621T), and ubiquitin (P4D1) mouse monoclonal antibody (for WB, 1:1,000; catalog no. 3936T) were obtained from Cell Signaling Technology (Massachusetts, USA). Rabbit recombinant monoclonal UBC linkage-specific K27 antibody (for WB, 1:3,000; catalog no. Ab181537) and rabbit recombinant monoclonal UBC4 antibody (which recognizes UBE2D2 for WB, 1:5,000; catalog no. ab155088) were obtained from Abcam (Cambridge, UK). Mouse monoclonal antibody against PRRSV M protein (for IFA, 1:1,000; for WB, 1:2,000) was kindly provided by Professor Zhijun Tian.

### Plasmids

The human OTUB1 gene (GenBank accession no. NM_017670) was amplified from HEK-293T cell cDNA using PrimeSTAR HS DNA polymerase (Takara, Dalian, China) and cloned into the VR1012-MYC vector to generate pMYC-OTUB1. Truncated mutants of OTUB1, pMYC-OTUB1^1–85^ (encoding amino acids 1–85) and pMYC-OTUB1^86–271^ (encoding amino acids 86–271), were constructed using pMYC-OTUB1 as the template. Site-directed mutagenesis was performed to generate catalytically inactive (pMYC-OTUB1-C91A) and E2-binding-deficient (pMYC-OTUB1-D88A) mutants using the Fast Mutagenesis System (TransGen, Beijing, China). The ORF6 gene of PRRSV L251 strain was synthesized by Sangon Biotech after codon optimization and cloned into the pCl-3×FLAG vector to construct pFLAG-M. Truncated M protein plasmids, pFLAG-mCherry-M^1–91^ (amino acids 1–91) and pFLAG-mCherry-M^92–174^ (amino acids 92–174), were generated by PCR amplification of pFLAG-M. The ORF5 gene of the L251 strain was amplified from cDNA of L251-infected MARC-145 cells and inserted into the VR1012 vector to obtain pFLAG-GP5. ORF6 genes of PRRSV HBBX and VR2332 strains were amplified from cDNA of infected MARC-145 cells and inserted into the pCl-3×FLAG vector. LNWK96 (GenBank accession no. MG860516.1) and LV (GenBank accession no. NC_043487.1) were synthesized by Sangon Biotech and cloned into the pCl-3×FLAG vector. Plasmids encoding HA-tagged ubiquitin (pHA-Ub) were preserved in our lab. Using VR1012-OTUB1-MYC as a template, the OTUB1-MYC sequence was amplified and connected to the pLVX-EF1α-IRES-Puro vector through homologous recombination to construct pLVX-EF1α-IRES-Puro-OTUB1-MYC. With pLK0.1 as the vector, the OTUB1-specific shRNA sequence was introduced through site-directed mutagenesis to construct pLK0.1-shOTUB1-1 and pLK0.1-shOTUB1-2. All plasmids were verified by Sanger sequencing (Sangon Biotech).

### Construction of stable OTUB1-knockdown cell lines

Lentiviral particles were produced by co-transfecting HEK-293T packaging cells with the transfer plasmid pLKO.1 carrying either a non-targeting control shRNA (shControl) or OTUB1-specific shRNAs (shOTUB1) together with the packaging plasmid psPAX2 and the envelope plasmid pMD2.G using polyethylenimine (PEI). The viral supernatant was harvested at 60 h post-transfection, filtered through a 0.45 μm membrane filter (Millipore, USA) and used to transduce HEK-293T cells (46). At 24 h post-transduction, cells were selected with 1 μg/mL puromycin (Yeasen, China) for 5 days to establish stable polyclonal populations. Knockdown efficiency was confirmed by Western blot analysis. The target sequences for the shRNAs are shown in Table S1.

### Construction of stable overexpressing OTUB1 cell lines

Lentiviral particles were produced by co-transfecting HEK-293T packaging cells with the transfer plasmid pLVX-EF1α-IRES-Puro carrying OTUB1 gene together with the packaging plasmid psPAX2 and the envelope plasmid pMD2.G using PEI. The viral supernatant was harvested at 60 h post-transfection, filtered through a 0.45 μm membrane filter (Millipore, USA) and used to transduce MARC-145 cells (46). At 24 h post-transduction, cells were selected with 2 μg/mL puromycin (Yeasen, China) for 5 days to establish stable polyclonal populations. Overexpression efficiency was confirmed by immunofluorescence assay and Western blot analysis.

### RNA interference

Short interfering RNAs (siRNAs) targeting monkey OTUB1 (siOTUB1) and negative control siRNA (siControl) were synthesized by Sangon Biotech (Shanghai, China). HEK-293T cells, MARC-145 cells, and PAMs were transfected with siRNA using Lipofectamine RNAiMAX (Invitrogen, USA) according to the manufacturer’s standard protocol. The efficiency of OTUB1 knockdown was confirmed by RT-qPCR and Western blot. The target sequences for the siRNAs are shown in Table S1.

### RT-qPCR

Total RNA was extracted from cells using the Total RNA Kit (TransGen Biotech, China). RNA quality was verified by Nano-300 spectrophotometer (Hangzhou Allsheng Instrument, China). The gDNA removal and first-strand cDNA synthesis were performed using EasyScript One-Step gDNA Removal and cDNA Synthesis SuperMix (TransGen Biotech, China). qPCR was conducted on a Bio-Rad CFX96 system (Hercules, USA) using Taq SYBR Green qPCR Premix (Yugong Biotech, China). Glyceraldehyde-3-phosphate dehydrogenase was used as an endogenous control for normalization. The target sequences for the qPCR are shown in Table S2.

### Immunoprecipitation and Western blot

For immunoprecipitation, cells were transfected with the indicated plasmids for 36 h, then lysed in IP lysis buffer (25 mM Tris-HCl, pH 7.4, 150 mM NaCl, 1 mM EDTA, 1% Nonidet P-40, 5% glycerol) supplemented with protease inhibitor cocktail. Cell lysates were centrifuged at 12,000 × *g* for 10 min at 4°C, and supernatants were incubated with the corresponding antibody overnight at 4°C with gentle rotation. Subsequently, Protein A/G magnetic beads (Beaver, China) were added and incubated for another 4 h at 4°C. Beads were washed five times with washing buffer (50 mM Tris-HCl, pH 7.4, 50 mM NaCl, 0.1% Tween-20) and eluted by boiling in 4× SDS sample buffer for 10 min.

For Western blot, total cell lysates or IP eluates were separated by SDS-PAGE and transferred to nitrocellulose membranes (Cytiva, USA). Membranes were blocked with 5% non-fat milk in TBST (20 mM Tris-HCl, pH 7.5, 150 mM NaCl, 0.1% Tween-20) for 1 h at room temperature, then incubated with primary antibodies overnight at 4°C. After three washes with TBST, membranes were incubated with HRP-conjugated secondary antibodies for 1 h at room temperature. Signals were detected using an Enhanced Chemiluminescence Kit (Proteintech, China) and visualized with a ChemiScope 6200Touch Chemical Luminescence Imaging System (Clix, China). The intensities of protein bands were quantified using ImageJ software (National Institutes of Health, USA).

### Immunofluorescence assay

Cells were fixed with a 1:1 mixture of methanol and acetone (pre-cooled to −20°C) for 30 min at room temperature. Subsequently, cells were incubated with primary antibodies at 37°C for 1 h. Following PBS washes, cells were incubated with fluorescently labeled secondary antibodies at 37°C in the dark for 1 h. The nuclei were stained with Hoechst 33342 (Solarbio, China) for 10 min at room temperature. After a final PBS wash, fluorescence signals were observed. The co-localization analysis was performed using the Leica TCS SP8 laser confocal microscope (Leica Microsystems, Germany), while routine staining was visualized with a fluorescence microscope (BioTek Lionheart LX, BioTek Instruments).

### Flow cytometry

MARC-145 cells or PAMs were infected with rL251-eGFP. At 24 h or 36 h post-infection, cells were digested with 0.25% trypsin-EDTA, centrifuged at 1,000 × *g* for 5 min, and resuspended in PBS. The percentage of eGFP-positive cells was measured using a TanCyte0208HTR flow cytometer (Tuan Biotechnology, China) and analyzed with SenseFlow software (Tuan Biotechnology, China).

### Statistical analysis

Data were analyzed using GraphPad Prism software (GraphPad Software, San Diego, USA) and presented as mean ± standard deviation. Comparisons between two groups were performed using an unpaired two-tailed Student’s *t*-test, and multiple group comparisons were analyzed by one-way analysis of variance followed by Tukey’s *post hoc* test. Statistical significance was defined as *P < 0.05, **P < 0.01, ***P < 0.001, and ****P < 0.0001.

## Data Availability

The data supporting the findings of this study are included in the article and supplemental material.

## SUPPLEMENTAL MATERIAL

Supplemental material jvi.01868-25-s0001.docx Fig. S1 and S2; Tables S1 and S2.

ASM does not own the copyrights to Supplemental Material that may be linked to, or accessed through, an article. The authors have granted ASM a non-exclusive, world-wide license to publish the Supplemental Material files. Please contact the corresponding author directly for reuse.
