# Supplementary material for: OTUB1 stabilizes PRRSV matrix protein through a non-canonical deubiquitination mechanism to promote viral replication
Source: J Virol. 2026 Mar 31;100(4):e01868-25. doi: 10.1128/jvi.01868-25 (PMC13098273; doi:10.1128/jvi.01868-25)
Supplement: Supplemental material — Fig. S1 and S2; Tables S1 and S2. [file jvi.01868-25-s0001.docx]

**Supplementary Material**

**
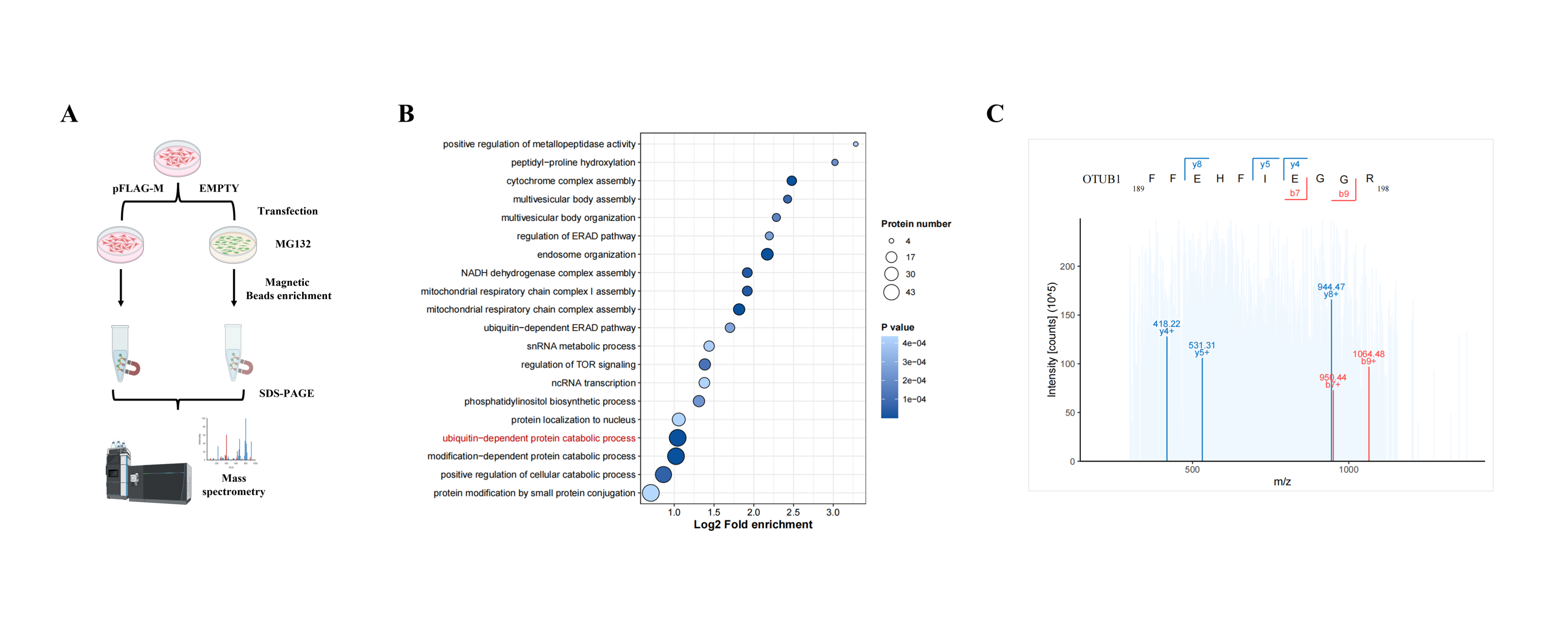
**

**Fig. S1. Identification of M-interacting host proteins by LC-MS.** (A) Schematic workflow of the mass spectrometry identification process. HEK-293T cells transfected with pFLAG-M (or EV). Lysates were subjected to IP with anti-FLAG antibody followed by LC-MS analysis. (B) KEGG pathway enrichment analysis of the identified M-interacting proteins. (C) Representative MS/MS spectra of the unique OTUB1 peptide identified.

**
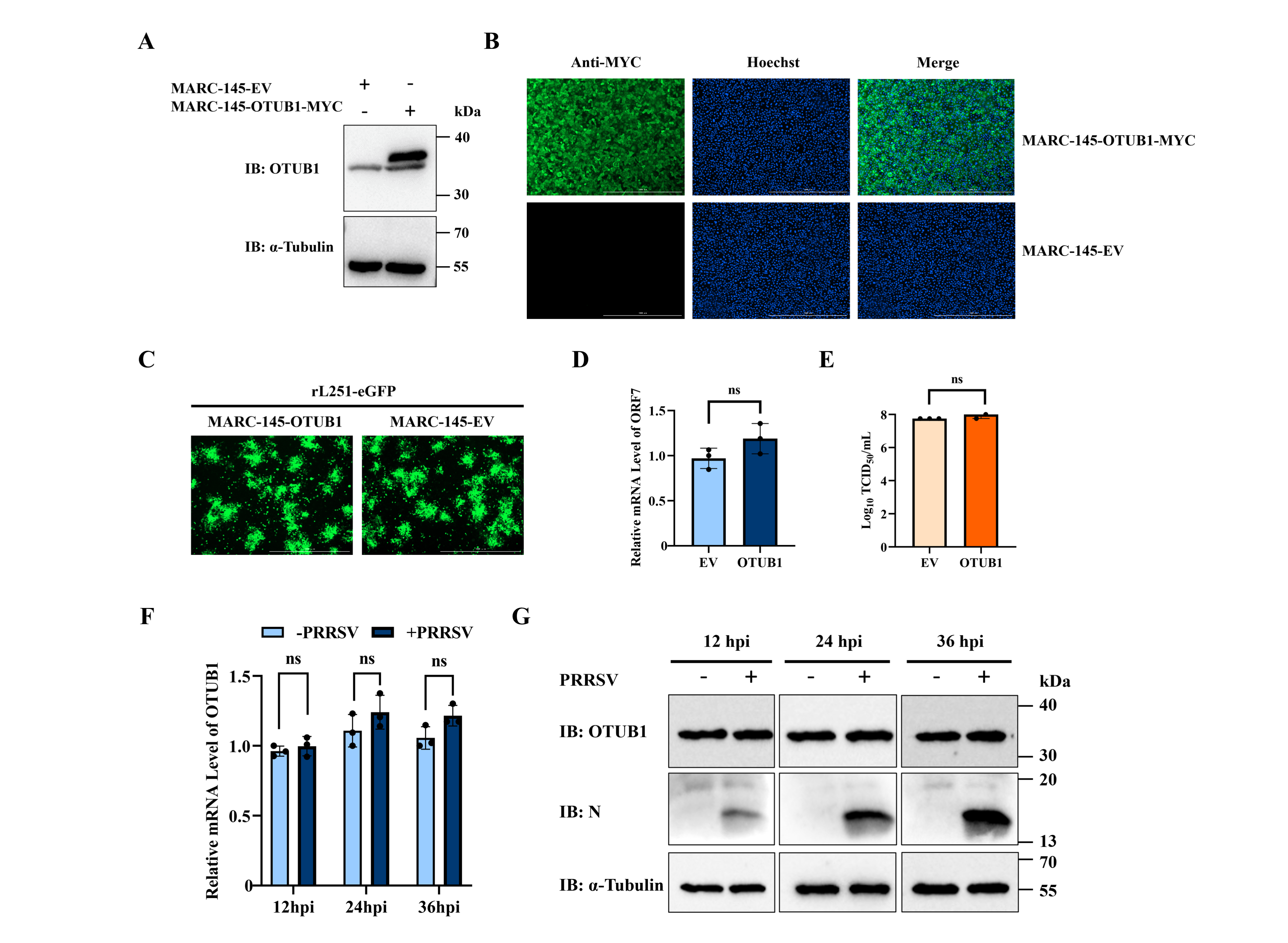
**

**Fig. S2. Effect of OTUB1 overexpression on PRRSV replication and expression dynamics of endogenous OTUB1.** (A and B) Validation of stable OTUB1-overexpressing MARC-145 cells. Cell lines generated by lentiviral transduction were analyzed by Western blot (A) and IFA (B) to confirm OTUB1 expression. (C) Effect of OTUB1 overexpression on reporter virus replication. MARC-145-OTUB1 or control cells were infected with rL251-eGFP (MOI = 0.5). Fluorescence images were captured at 48 hpi. (D and E) Effect of OTUB1 overexpression on WT PRRSV replication. Cells were infected with L251 (MOI = 0.5). Intracellular viral mRNA levels (D) and viral titers in the supernatant (E) were determined at 48 hpi by RT-qPCR and TCID50, respectively. (F and G) Expression patterns of OTUB1 during PRRSV infection. MARC-145 cells were infected with PRRSV (MOI = 0.5) or mock-infected. Samples were collected at 12, 24, and 36 hpi to analyze OTUB1 mRNA (F) and protein (G) levels.

**Supplemental Table 1. Primers used in this study**

| **shRNA/siRNA Name** | **Sequence** | **Description** |
| --- | --- | --- |
| OTUB1-1 | 5′-CCGAAGGTGTTAACTGTCTGGCCTA-3′ | shRNA1 for OTUB1 (Human) |
| OTUB1-2 | 5′-CATCATGGCTCAGCAGGACCGAATT-3′ | shRNA2 for OTUB1 (Human) |
| No Target-s | 5′-UUCUCCGAACGUGUCACGU/dT//dT/-3′ | Control siRNA |
| No Target-a | 5′-ACGUGACACGUUCGGAGAATT/dT//dT/-3′ |  |
| OTUB1-s | 5′-GCAAGAGAUUGCUGUGCAGAA/dT//dT/-3′ | siRNA for OTUB1  (Human/Chlorocebus sabaeus) |
| OTUB1-a | 5′-UUCUGCACAGCAAUCUCUUGC/dT//dT/-3′ |  |
| UBE2E1-s | 5′-CGUGUAUGAGGGUGGUGUA/dT//dT/-3′ | siRNA for UBE2E1 (Human) |
| UBE2E1-a | 5′-UACACCACCCUCAUACACG/dT//dT/-3′ |  |
| UBE2N-s | 5′-CCAGAUGAUCCAUUAGCAA/dT//dT/-3′ | siRNA for UBE2N (Human) |
| UBE2N-a | 5′-UUGCUAAUGGAUCAUCUGG/dT//dT/-3′ |  |
| UBE2D1-s | 5′-GACUCCUGAUAGCGCAUAU/dT//dT/-3′ | siRNA for UBE2D1 (Human) |
| UBE2D1-a | 5′-AUAUGCGCUAUCAGGAGUC/dT//dT/-3′ |  |
| UBE2D2-s | 5′-CUCUGUUGUGUGAUCCCAA/dT//dT/-3′ | siRNA for UBE2D2 (Human) |
| UBE2D2-a | 5′-UUGGGAUCACACAACAGAG/dT//dT/-3′ |  |
| UBE2D3-s | 5′-GAGAUUGCACGGAUCUAUA/dT//dT/-3′ | siRNA for UBE2D3 (Human) |
| UBE2D3-a | 5′-UAUAGAUCCGUGCAAUCUC/dT//dT/-3′ |  |

**Supplemental Table 2. Primers used in this study**

| **Primer Name** | **Sequence** | **Description** |
| --- | --- | --- |
| OTUB1-Fw1 | 5′-TCGAGCAGGTAGAGAAGCAG-3′ | RT-qPCR for OTUB1  (Chlorocebus sabaeus) |
| OTUB1-Rv1 | 5′-AAGGTAGTCGGAGGTGCTCT-3′ |  |
| OTUB1-Fw2 | 5′-TCGAGCAGGTGGAGAAGCAG-3′ | RT-qPCR for OTUB1 (Sus scrofa) |
| OTUB1-Rv2 | 5′-CAGGTAGTCCGAGGTGCTCT-3′ |  |
| GAPDH-Fw1 | 5′-GTCTCCTCTGACTTCAACAGCG-3′ | RT-qPCR for GAPDH (Human/Chlorocebus sabaeus) |
| GAPDH-Rv1 | 5′-ACCACCCTGTTGCTGTAGCCAA-3′ |  |
| GAPDH-Fw2 | 5′-ATGACATCAAGAAGGTGGTG-3′ | RT-qPCR for GAPDH (Sus scrofa) |
| GAPDH-Rv2 | 5′-CGTACCAGGAAATGAGCTTG-3′ |  |
| N-Fw | 5′-GAAGAAAAACCCGGAGAA-3′ | RT-qPCR for N |
| N-Rv | 5′-AACTAAACTCCACAGTGTAA-3′ |  |
| M-Fw | 5′-TTCATCACCTCCAGATGCCG-3′ | RT-qPCR for M |
| M-Rv | 5′-AATGTGCCGTTGACCGTAGT-3′ |  |
| UBE2E1-Fw1 | 5′-CCTCCTTTCTATCTGCTCACTTC-3′ | RT-qPCR for UBE2E1 (Human) |
| UBE2E1-Rv1 | 5′-GTAGCGTATCTCTTGGTCCACTG-3′ |  |
| UBE2N-Fw1 | 5′-TGATGTAGCGGAGCAGTGGAAG-3′ | RT-qPCR for UBE2N (Human) |
| UBE2N-Rv1 | 5′-GGAGGAAGTCTTGGCAGAACAG-3′ |  |
| UBE2D1-Fw1 | 5′-GCGCATATCAAGGTGGAGTCTTC-3′ | RT-qPCR for UBE2D1 (Human) |
| UBE2D1-Rv1 | 5′-CCATTGTGACCTCAGAATATCGAG-3′ |  |
| UBE2D2-Fw1 | 5′-CTACGATCACAGTGGTCTCCAG-3′ | RT-qPCR for UBE2D2 (Human) |
| UBE2D2-Rv1 | 5′-CGAGCAATCTCAGGCACTAAAGG-3′ |  |
| UBE2D3-Fw1 | 5′-GATCACAGTGGTCGCCTGCTTT-3′ | RT-qPCR for UBE2D3 (Human) |
| UBE2D3-Rv1 | 5′-AGATCCGTGCAATCTCTGGCAC-3′ |  |
